# Tarkhineh as a new microencapsulation matrix improves the quality and sensory characteristics of probiotic *Lactococcus lactis* KUMS-T18 enriched potato chips

**DOI:** 10.1038/s41598-021-92095-1

**Published:** 2021-06-15

**Authors:** Amir Kiani, Yousef Nami, Shahab Hedayati, Mehdi Jaymand, Hadi Samadian, Babak Haghshenas

**Affiliations:** 1 Regenerative Medicine Research Center (RMRC), Health Technology Institute, Kermanshah University of Medical Sciences, Kermanshah, 67146 Iran; 2 Department of Food Biotechnology, Branch for Northwest and West Region, Agricultural Biotechnology Research Institute of Iran, Agricultural Research, Education and Extension Organization (AREEO), Tabriz, Iran; 3 Students Research Committee, Kermanshah University of Medical Sciences, Kermanshah, Iran; 4 Nano Drug Delivery Research Center, Health Technology Institute, Kermanshah University of Medical Sciences, Kermanshah, Iran

**Keywords:** Microbiology, Applied microbiology

## Abstract

In the present study, probiotic potato chips containing a newly isolated probiotic *Lactococcus lactis* KUMS-T18 strain were produced by using a simple spraying method and then enhancing the stability, survival rate, and sensory characteristics of product during storage at 4 °C and 25 °C was examined for four months. Based on the results, *Lactococcus lactis* KUMS-T18 isolated from traditional Tarkhineh as a safe strain had high tolerance to low pH and high bile salt, anti-pathogenic activity, hydrophobicity, adhesion to human epithelial cells, auto- and co-aggregation, cholesterol assimilation and antibiotic susceptibility. Meanwhile, by micro-coating the probiotic cells in Tarkhineh formulations, elliptical to spherical shape (460–740 µm) probiotic drops were produced. The results revealed that potato chips produced with turmeric and plain Tarkhineh during storage at 4 °C, had excellent protection abilities for probiotic cells with about 4.52 and 3.46 log decreases in CFU/g respectively. On the other hand, probiotic potato chips, compared to non-probiotic and commercial potato chips, showed the criteria of probiotic products such as excellent quality and superior sensory characteristics. In summary, this study proved that probiotic *Lactococcus lactis* KUMS-T18 strain covered by Tarkhineh formulations as protective matrix has high potential to be used in the production of probiotic potato chips.

## Introduction

Probiotics are scientifically defined as non-pathogenic microorganisms that, if used consistently and in sufficient doses, have beneficial effects on consumer health. Probiotics mainly belong to the lactic acid bacteria (LAB) group. *Lactobacillus*, *Bifidobacterium*, *Lactococcus*, and *Enterococcus* are the most popular probiotic strains belonging to the LAB group that have been licensed for use in food products^1^. Traditional fermented dairy products such as Tarkhineh, which are produced and consumed in different parts of Iran, especially in the west of the country (Kermanshah province), can be considered as the main source of native and useful probiotic strains. Tarkhineh, which can be stored for a long time (1–2 years), is prepared from a mixture of yogurt drink or diluted yogurt, crushed wheat and prepared by adding spices and salt^2^. This product has a high content of vitamins, amino acids and free minerals^3,4^. On the other hand, Tarkhineh is a rich source of various types of probiotics belonging to the LAB group, especially Lactobacilli and Enterococci^5,6^.

Proven therapeutic effects of probiotics include increasing nutrient absorption, lowering blood cholesterol, reducing the severity of irritable bowel syndrome, treating antibiotic-related diarrhea, and their anti-cancer properties^7^. Therefore, due to the healing effects of these beneficial microorganisms, in recent years, extensive research has been conducted on the production and commercialization of appetizers containing probiotics such as potato chips^8,9^.

Potato chips as a high-consumption nutritious snack contain large amounts of starch, fat, fiber, as well as essential micronutrients such as potassium, sodium, and carotenoids^10,11^. But its high fat content, negatively affects the shelf life and taste of the final product. Also, due to the oxidation process of lipids during storage, the sensory acceptability of the potato chips is reduced. On the other hand, because of high surface to volume ratio, the oxidative degradation process in the stored product is high^12^. Therefore, to overcome the mentioned problems, various natural and chemical antioxidants have been evaluated to add to potato chips^13^.

Probiotics as natural antioxidants reduce the amount of fat oxidation in the processed products. And therefore, they prevent unpleasant flavor and odor in nutritious products such as potato chips^14,15^. But the main challenge for using probiotics is to maintain the effective dose of live bacteria at various heat/moisture conditions in the final product and during the storage period^16^. Lyophilization is the most common way for the stability of probiotics in food products. However, probiotics are sensitive to the loss of cell water during the lyophilization process and also this process has a low efficiency during long storage. This method has been widely used to stabilize probiotic bacteria in dairy and non-dairy products such as apple snacks^17^, soy bars^18^, sausages^19^, chocolate^20^ and many dairy products^21^. However, applying this technique for use in potato chips is not very effective^22^. Therefore, covering probiotic cells inside the nutrient and protective matrix can be a solution^23^.

In this study, potato chips were selected due to high consumption in Iran. On the other hand, probiotic strains isolated from Tarkhineh were used to produce probiotic potato chips due to their proven beneficial effects and compatibility with the covering matrix. Also, the potential of using spraying method for two different formulations of Tarkhineh paste (plain Tarkhineh and Tarkhineh turmeric) as a coating matrix for probiotic cells during long storage time was investigated. The small-scale spraying method is simple, low cost, and has a high survival rate of viable cells during the process. In addition, by using the right concentration of coatings materials, small probiotic drops can be produced to be added to potato chips. Probiotic evaluations, morphological characteristics, survival rates and sensory assessments of probiotic cells covered with Tarkhineh paste in potato chips were investigated in this research. Therefore, the primary goal was to isolate, evaluate and select the most appropriate probiotic strain from traditional Tarkhineh and the secondary goal was to investigate the effect of adding probiotic Tarkhineh drops on the quality of potato chips during storage time.

## Materials and methods

### Sampling and culture conditions

All experiments in this study were performed in accordance with the relevant guidelines/regulations of regional ethics committee in biomedical research of Kermanshah University of Medical Sciences with approval ID: IR.KUMS.REC.1398.1072.

Sixty samples of traditional Tarkhineh were collected from the domestic producers in Kermanshah province in Iran. These samples were transferred to the laboratory and stored at refrigerator 4 °C. The samples were enriched by adding 1% volume to 50 mL of sterile de Man Rogosa Sharpe (MRS) broth and incubated at 37 °C for 48 h. 100 µL of each sample were spread over solidified MRS medium for enumeration and incubated 24 h at 37 °C. Then, the single colonies with distinct morphology were picked and subjected to initial morphological and biochemical tests^24^.

### Acid and bile tolerance

To assess acid tolerance, the MRS broth with pepsin enzyme (3 mg/mL) was used as a medium. The pH of broth was adjusted to pH 2.5 with 1.0 N HCl and broth (pH 7.0) was used as a control. Further, the broth was inoculated 3 h and the optical density (OD) was measured at 600 nm (data not shown). Viable counts were also considered for selected strains (Table 1).

**Table 1:** Re-screening results and survival rates (%) of selected LAB after 3 h incubation at pH 2.5 and 4 h incubation at 0.3% bile salt.

Isolates	Final counts (log CFU/mL) after 3 h incubation at pH 2.5					Final counts (log CFU/mL) after 4 h incubation at 0.3% bile salt					
	0 h	1 h	2 h	3 h	SR (%)	0 h	1 h	2 h	3 h	4 h	SR (%)
T3	9.743	9.418	7.493	7.112	73	9.743	9.393	8.618	8.242	7.989	82
T18	9.146	9.019	8.684	8.440	92	9.146	9.133	9.087	9.071	9.054	99
T29	9.051	8.489	6.981	6.426	71	9.051	8.824	7.615	7.497	7.241	80
T39	9.614	9.008	7.232	6.922	72	9.614	9.424	8.197	7.593	7.307	76
T46	9.819	9.403	8.142	7.953	81	9.819	9.724	9.381	9.179	8.935	91

*SR* Survival rate.

The ability of strains to grow in presence of bile salt was measured. This was performed using 0.3% w/v oxgall and Control was maintained using MRS broth. The samples were then inoculated at 37 °C for 4 h and OD_620_ of samples was measured to check the viability of cells^25^. Finally, the acid and bile tolerance was estimated by determining the survival rate by following equation:

$$ {\text{Survival }}\,{\text{Rate }}\left( \%  \right):{\text{ }}\,\left[ {{\text{OD }}\left( {{\text{After}}\,{\text{ treatment}}} \right)/{\text{OD }}\left( {{\text{Before}}\,{\text{ treatment}}} \right)} \right]{\text{ }} \times {\text{ 1}}00{\text{ }}\% . $$

### Hemolytic activity

The overnight grown strains were streaked on blood agar plate and incubated at 37 °C for 24–48 h. The plates were observed for the formation of any β-hemolysis (clear haloes) or α-hemolysis (greenish haloes) and γ-hemolysis (no haloes) around the colonies (Table 2)^1^.

**Table 2:** Results of hydrophobicity, auto-aggregation, cholesterol assimilation rate, and hemolysis.

Strains	Hydrophobicity (%)	Auto-aggregation (%)	Cholesterol assimilation rate (%)	Hemolysis
T3	–	–	–	α
T18	63 ± 0.9^a^	79 ± 1.3^a^	76 ± 2.1^a^	γ
T29	51 ± 0.7^b^	54 ± 1.1^b^	30 ± 0.9^b^	γ
T46	–	–	–	α
T39	43 ± 1.0^c^	44 ± 1.2^c^	23 ± 1.2^c^	γ

Values are mean ± standard deviation of triplicates.

^a–c^Means in the same column with same lowercase letters are not differed significantly (*p* ≤ 0.05).

### Inhibitory effects against pathogens

The agar well diffusion technique was performed to measure the antagonistic activity of the isolated strains against some foodborne and clinically important human pathogens listed in Table 3. About 1.5 × 10^8^ CFU/mL (half McFarland) of mentioned pathogens was cultured on Mueller–Hinton agar and incubated at 4 °C for 2 h. Then, the wells were cut on inoculated medium and filled with 100 µL of filtered supernatant (overnight cultured) of selected isolates and incubated overnight at 37 °C. Finally, the inhibition zone was measured by digital caliper^25^.

**Table 3:** The inhibitory effect of selected LAB strains against pathogens. Values shown are means ± standard deviations (n = 3).

Isolates	Diameter of inhibition zone (mm)							
	Indicator pathogens							
	*Y. enterocolitica*	*S. mutans*	*E. coli*	*S. aureus*	*B. subtilis*	*L. monocytogenes*	*K. pneumoniae*	*S. flexneri*
T18	11.8 ± 0.4^a^	11.7 ± 0.8^a^	10.6 ± 1.0^a^	11.4 ± 0.7^a^	13.3 ± 0.5^a^	12.7 ± 1.0^a^	11.0 ± 0.4^a^	12.9 ± 0.4^a^
T29	9.6 ± 0.6^b^	8.9 ± 0.4^c^	9.6 ± 0.7^a^	9.6 ± 0.8^b^	10.8 ± 0.8^b^	11.0 ± 0.5^b^	8.6 ± 0.9^b^	8.9 ± 0.5^b^
T39	8.6 ± 0.7^b^	10.6 ± 0.7^b^	9.4 ± 0.7^a^	11.6 ± 0.8^a^	9.2 ± 0.4^c^	9.6 ± 0.8^c^	8.4 ± 1.6^b^	8.9 ± 0.5^b^

*Values are mean ± standard deviation of triplicates.

S (strong *r* ≥ 20 mm), M (moderate *r* < 20 mm and > 10 mm), and W (weak ≤ 10 mm).

^a–d^Means in the same row with different lowercase letters differed significantly (*p* ≤ 0.05).

#### Characterization of antimicrobial substances

The nature of anti-pathogenic substances in bacterial extracts was evaluated based on method described by Nami et al.^26^ with some modifications. The cell-free supernatant (CFS) was obtained by centrifugation at 10,000 rpm for 20 min at 4 °C. The pH was adjusted to 6.2 and treated with 1 mg/mL proteinase K (protein nature assessment) and catalase (hydrogen peroxide evaluation) for 2 h at 37 °C. Finally, anti-pathogenic activity was measured by well diffusion method.

### Antibiotic susceptibility profiles

The disc diffusion method was performed for evaluation of the susceptibility of strains to some high-consumption and clinically important antibiotics listed in Table 4. For this purpose, the samples were spread over the solidified MRS medium and antibiotic disks were placed on them and incubated overnight at 37 °C and placed antibiotic disks on them, the diameter of inhibition around disks were measured by digital caliper^27^.

**Table 4:** Antibiotic susceptibility profiles of isolated LAB.

Isolated strains	Antibiotics susceptibility zone of inhibition (mm)								
	CFM	AZM	AMX	D	SXT	CP	CN	AMC	V
T18	22 (S)	14 (I)	35 (S)	21 (S)	25 (R)	0 (R)	32 (S)	39 (S)	0 (R)
T29	16 (I)	16 (I)	30 (S)	21 (S)	23 (R)	0 (R)	35 (S)	38 (S)	0 (R)
T39	24 (S)	19 (S)	32 (S)	25 (S)	25 (R)	0 (R)	35 (S)	37 (S)	0 (R)

Cefixime results based on R ≤ 15 mm; I: 16–18 mm; S ≥ 19 mm. Azithromycin results based on R ≤ 13 mm; I: 14–17 mm; S ≥ 18 mm. Amoxicillin results based on R ≤ 18 mm; I: 19–21 mm; S ≥ 22 mm. Doxycycline results based on R ≤ 10 mm; I: 11–13 mm; S ≥ 14 mm. Trimethoprim sulfamethoxazole results based on R ≤ 25 mm; I: 26–29 mm; S ≥ 30 mm. Ciprofloxacin results based on R ≤ 15 mm; I: 16–20 mm; S ≥ 21 mm. Cephalexin results based on R ≤ 14 mm; I: 15–17 mm; S ≥ 18 mm.

Amoxicillin-clavulanic acid results based on R ≤ 13 mm; I: 14–17 mm; S ≥ 18 mm. Vancomycin results based on R ≤ 14 mm; I: 15–16 mm; S ≥ 17 mm.

Performance Standards for Antimicrobial Susceptibility Testing, from Clinical and Laboratory Standards Institute, Twenty-Third Informational Supplement, Wayne, PA (CLSI 2013).

*CFM* cefixime, *AZM* azithromycin, *AMX* amoxicillin, *D* doxycycline, *SXT* trimethoprim sulfamethoxazole, *CP* ciprofloxacin, *CN* cephalexin, *AMC* amoxicillin-clavulanic acid, *V* vancomycin.

### Cell surface hydrophobicity

The ability of strains to adhere to hydrocarbons was determined by modified method of Nami et al.^28^. Overnight cultures of selected strains were harvested by centrifugation at 10,000 rpm for 10 min and the pellets (10^8^ CFU/mL) were suspended in 3 mL of phosphate buffered saline (PBS) and initial absorbance at 600 nm (A_0_) was measured. Then, each bacterial suspension was allowed to come in contact with 1 mL of xylene (Merck, Germany) by whirling on vortex for 2 min. The phases were allowed to separate by decantation at 37 °C for 1 h and the absorbance of aqueous phase was measured (A_1_). The test was done in triplicate and expressed as Hydrophobicity (%) = (1 − A_1_/A_0_) × 100.

### In-vitro cell adhesion assay

Adhesion ability of selected strains to human epithelial cells was determined by using CaCO_2_ cell line. The CaCo2 cell lines were grown in a controlled atmosphere of 5% CO_2_ AT 37 °C in RPMI-1640 medium (Sigma) supplemented with 10% heat-inactivated fetal bovine serum and 50 unit/mL penicillin–streptomycin. For the bacterial adhesion assay, CaCO_2_ cells were seeded on glass cover-slips that were placed in 6-well tissue culture plates and after 24 h of incubation at 37 °C (5% CO_2_ and 95% air), the monolayers were washed twice with sterile PBS (pH 7.4) and 10 mL of bacterial suspension (1 × 10^7^ CFU/mL) was added to each plate. The inoculated plates were incubated at 37 °C for 2 h followed by washing 3 times with PBS (pH 7.4) to remove non-adherent bacteria. The adherent bacteria were detached by utilizing a solution of trypsin–EDTA (0.05%) and resuspended in 10 mL of saline solution. Then, serial dilutions of bacteria were cultured on MRS agar and incubated at 37 °C for 24–48 h. The percentage of adhesion was measured by comparison of the number of adhered cells to the total cells of the examined bacterial suspension^1^. Each adherence assay was performed in triplicate, and the values were expressed as Mean value Standard Deviation (SD).

### Cholesterol assimilation

Cholesterol reduction ability of strains was measured using o-phthaldehyde method described by Miremadi et al.^29^. The strains were inoculated into MRS broth supplemented with water soluble cholesterol (polyoxyethanylcholesterylsebacate; Sigma) with a concentration of 150 μg/mL and 0.3% bile oxgall and incubated 20 h at 37 °C. After this period, the cell pellet was harvested by centrifugation at 4300×*g* for 15 min, and the remaining cholesterol in the upper layer was measured by o-phthaldehyde method.

### Auto-aggregation and co-aggregation ability

The auto-aggregation ability of strains was performed by method of Angmo et al.^30^. The following equation (% = 1 − (A_t_/A_0_) × 100) was used to determine the percentage of auto-aggregation.

Co-aggregation of selected strains against four pathogens was performed based on method described by Zuo et al.^31^. The percentage of co-aggregation was determined based on following equation:

$$ \%  = \frac{{{\text{A}}_{0}  - {\text{A}}_{{\text{t}}} }}{{{\text{A}}_{{\text{t}}} }}{\text{~}} \times 100, $$

where A_0_ represents absorbance at t = 0 and A_t_ represents absorbance at time t^31^.

### Potato chips preparation

Potato (*Solanum tuberosum* var. *Ramoae*) was prepared from the local market, Kermanshah, Iran. Soybean oil was obtained from of Nazgol Company (Mahidasht, Kermanshah, Iran). The potato tubers were washed well and after peeling, they were cut into thin slices (1–2 mm). Then, for more crispness, they were soaked in CaCl_2_ solution (1%) for 15 min. After the refined soybean oil reached a temperature of 200 ℃ in a stainless steel pan, the potato slices (dimensions > 5 cm × 5 cm) were fried for 5–7 min and cooled after taking the extra oil ^23^.

### Preparation of probiotic cell culture

The probiotic strains were enriched by anaerobic growth of 100 µL respective stock cultures in 15 mL MRS medium for 24 h at 37 °C. The cell pellets were harvested by centrifugation at 4000 rpm for 10 min at 4 °C, washed and re-suspended in phosphate buffer (pH 7.4). Prior to the *preparation* step, they were counted in MRS agar using pour plate method three times. The equal volume of the viable cell population was divided and used in the *preparation* step by different Tarkhineh blends^32^.

### Probiotic Tarkhineh preparation and chemical analysis

First, wheat semolina (500 g) was washed and soaked in diluted buttermilk (1.5 L) for 3–4 h, and salt (10 g), dried mint, and pennyroyal (5 g) were added. The mixture was divided into two separate pots which were placed on the heater for 2 h and stirred constantly until it boiled and becomes thick. Ten gram turmeric was added into one pots (turmeric Tarkhineh), and plain Tarkhineh was added into other pot. The solutions were stirred well until the buttermilk was absorbed into the wheat semolina and became relatively pasty. The flame was then extinguished, and after cooling, it was completely homogenized and finely crushed by a mixer. A certain amount of both plain and turmeric Tarkhineh was removed separately in 50 mL falcons and after autoclaving, they were inoculated with selected probiotic bacteria and then incubated at 37 °C until use. The difference between the weight of Tarkhineh paste before and after drying was considered as the percentage of moisture. The pH level of Tarkhineh paste was measured. The percentage of calcium and phosphorus was estimated by spectrophotometry^33^. The method described by Mashak et al.^33^ was used to determine the amount of protein, salt, ash and fat. Finally, the percentage of carbohydrates was obtained by reducing the total weight of solids from the weight of fat, salt, protein, and ash.

### Probiotic potato chips production, morphological analysis, and spraying efficiency

The potato slices were placed in a sterile environment in special polyethylene bags and then the probiotic Tarkhineh containing a certain number of LAB was sprayed on the surface of the potato slices in small drops by a handmade sprayer. The Tarkhineh paste containing probiotics was transferred through a sterile tank by a pump to the sprayer head at the end of the machine and turns into fine liquid droplets. Under the laminar flow cabinet, the droplets are sprayed on the surface of the potato chips and then dried^23^.

After potato chips production, the size of probiotic drops was measured using a laser diffraction particle size analyzer (Mastersizer 3000, Malvern Instruments, UK). The average size of the drops was estimated from the mean diameter of 50 drops obtained from each of the potato chips samples. The morphology of probiotic drops was investigated using a scanning electron microscopy (Hitachi SU3800). For better conductivity during imaging, samples were covered for 500 s with an ultrathin layer of gold (thickness of approximately 5–6 nm) by a sputter coater.

To determine the spraying efficiency, 50 mg of probiotic drops directly after spraying was disintegrated in 5 mL phosphate buffer (pH 7.4) at 37 °C for 20 min and subsequently the entrapped viable probiotic cells were counted by the pour plate technique in MRS agar. The spraying efficiency was calculated by the following equation:

$$ {\text{Spraying }}\,{\text{efficiency }}\left( {{\text{SE}}} \right){\text{ }} = {\text{ }}\left( {{\text{Log}}_{{{\text{1}}0}} {\text{N}}/{\text{Log}}_{{{\text{1}}0}} {\text{N}}_{0} } \right){\text{ }} \times {\text{1}}00, $$

where N is the number of entrapped viable probiotic cells and N_0_ displays the free viable probiotic cells before spraying^34^.

### Storage stability

Storage stability of bacteria was investigated during 120 days storage in vacuum packed polyethylene bags at room (25 °C) and refrigerated (4 °C) temperature in a dark place. The viability of cells was determined in seven different storage times (0, 20, 40, 60, 80, 100 and 120 days). During storage time, 1 g probiotic potato chips were dissolved in 10 mL sodium citrate solution (50 mM, pH 7.5) at room temperature by gentle shaking (100 rpm). The released probiotic cells were serially diluted 10 times by using saline solution, and then, 50 µL of aliquots was poured on the MRS agar and incubated for 24 h at 37 °C. The viable rates (%) of probiotic cells were calculated by utilizing the pour plate method in MRS agar. Meanwhile, potato chips containing lyophilized probiotic cells with 10% skim milk + 5% sucrose (SM) was used as controls^23,35^.

### Sensory evaluation

A trained panel of 15 students and staff of the faculty of pharmacy, Kermanshah University of medical sciences (Kermanshah, Iran) evaluated probiotic, non-probiotic and commercial potato chips samples in terms of various sensory characteristics such as color, smell, appearance, texture, taste, and general acceptance. Samples were evaluated for sensory evaluation using 9-point hedonic scale (from 9 to 1), while 9 was very likable and 1 was very unpleasant^36^.

These tests were approved by the IR.KUMS.REC.1398.1072. The informed consent was obtained from all participants in this research accordance with the declaration of Helsinki and a written consent was signed by all participants.

### Molecular characterization

The amplification of 16S-rRNA fragment was conducted by using a pair of LAB-specific universal primers (Hal6F/Hal6R) (F: 5′-AGAGTTTGATCMTGGCTCAG-3′ and R: 5′-TACCTTGTTAGGACTTCACC-3′). Amplified products were electrophoresed at a constant voltage of 70 V for one h and were visualized in a gel documentation system (Bio-Rad, USA). The DNA fragments with the size of 1500 bp indicate the right amplification. Finally, the amplified 16S-rRNA fragments were sequenced by Macrogene (Korea). The sequences were then analyzed using the BLAST program of the National Center for Biotechnology Information (http://www.ncbi.nlm.nih.gov/BLAST), in which the isolated bacteria were identified at strain levels.

### Statistical analysis

All experiments were designed based on a completely randomized design with three replications for each experimental group. The data were analyzed using ANOVA and Duncan statistical tests, also a significant difference (*p* ≤ 0.05) were considered for the means. In addition, SPSS statistics19 software was used in data analysis.

### Ethics

The study involving “production of optimized potato using probiotic bacteria isolated from Kermanshah indigenous dairy products” was reviewed and approved by Dr. Mahmood Reza Moradi (Chairman of the Academic/Regional Ethics Committee in Biomedical Research) and Dr. Farbod Najafi (Secretary of the Academic/Regional Ethics Committee in Biomedical Research) of Kermanshah University of Medical Sciences (Approval ID: IR.KUMS.REC.1398.1072).

## Results and discussion

### Bacterial morphological and biochemical analysis

Bacterial colonies with a whitish to creamy hemispherical form were isolated. As LAB bacteria, a total of 18 rod or spherical form bacteria that were catalase-negative and gram-positive were separated from the existing colonies and cultivated in a specified culture medium (MRS) under anaerobic conditions. These 18 isolates were chosen and subjected to further testing.

### Tolerance to low pH and high bile salt

Eighteen LAB strains were cultured under pH 2.5 for 3 h and 0.3% bile salt for 4 h. According to the preliminary results based on OD study (data not shown), 5 strains including T3, T18, T29, T39, and T46 had a survival rate of more than 76% and were therefore selected for re-screening test. The results of log CFU/mL for these five selected LAB strains is shown in Table 1. After 1 h of incubation under low pH and high bile salt conditions, slight decreases in log CFU (≤ 0.606) were observed. These results demonstrated that the cells showed high survival rates in the first hour of incubation. From 1 to 2 h, greater decreases in log CFU (0.046–1.925) were observed. Also, during this period, the tolerant strains showed a higher level of resistance than the sensitive strains. In the period of 2–3 h, the strains showed slight decreases in log CFU (≤ 0.604). Finally, from 3 to 4 h of incubation under bile salt condition, very slight decreases in log CFU (0.017–0.286) was observed among the cells. The moderate survival rates in harsh conditions from 71 to 82% were observed in T3, T29 and T39 strains. Strain T46 showed higher survival rates which were 81% at low pH and 91% at bile salt conditions. In addition, T18 strain had the best results and showed high tolerance to inverse conditions (92–99%).

To colonize the gastrointestinal tract and have health-promoting benefits, oral probiotics must get through the body's defense systems, which include low pH and bile salts. As a result, in vitro procedures with the same pH (2.5 for 3 h) and oxgall concentration (0.3 percent (w/ w) for 4 h) can be used to assess bacteria's tolerance to gastrointestinal circumstances^37^.

Optical density (OD) study at low pH and bile salts was used as a preliminary assessment to screen a large number of isolated bacteria. Although some of the tested strains survived well in acidic and bile environments, there was a large difference in the survival of the strains in artificial digestive juice. High tolerance of LAB to harsh conditions has been shown in other studies^38,39^. All 5 isolates displayed high tolerance to bile salt conditions, ranging from 4 to 10% higher than their low pH tolerance. Bile salts showed less lethal effects on bacterial cells than low pH. Numerous studies, as shown by our findings, suggested that bacteria are more resistant to bile salt environment due to their stress adaptation mechanisms in acidic conditions^37,40^. Based on the results, all five selected LAB strains retained their viability even after exposure to low pH and high bile salt environment. Notably, broad variations in survivals were observed at those conditions. As a result, these five isolates were selected for further analysis.

### Cell surface hydrophobicity test

Selected strains showed cell surface hydrophobicity ability with values above 43%. Strain T18 (63 ± 0.9) showed hydrophobic values significantly (*p* ≤ 0.05) higher than other tested strains. Strains T3 and T46 showed no hydrophobic characteristics (Table 2).

The capacity of bacteria to stick to the hydrophobic phase of the employed solvent is required to determine the ability of possible probiotic bacteria to cling to the intestine mucus^41^, preventing harmful bacteria from adhering to the intestine mucus 41 and infecting all available intestine walls^42^. Researchers previously hypothesized that differences in the quantity of surface protein production by bacteria could result in a wide range of cell surface hydrophobicity among LAB^43^. Several studies have also found a link between hydrophobicity and adhesion ability^44^.

### Inhibitory effects against pathogens

The antagonistic activities of 5 selected LAB against 8 pathogens are shown in Table 3. The results showed that three strains (T18, T29 and T39) displayed significant anti-pathogenic activities against indicator microorganisms and were able to inhibit the growth of all pathogens. Meanwhile*,* T46 exhibited moderate antagonistic activity and inhibited the growth of three pathogens including *Yersinia enterocolitica*, *Streptococcus mutans*, and *Klebsiella pneumoniae*. By contrast, T3 showed the weakest antagonistic activity and inhibited only one pathogen (*S. mutans*).

Strains T3 and T46 showed no anti-pathogenic properties after adjusting the pH of the bacterial extracts to 6.2. *L. monocytogenes*, *S. flexneri*, *B. subtilis*, and *K. pneumoniae* were also unaffected by strains T18, T29, and T39. As a result, the nature of inhibition of these isolates against the specified pathogens is determined to be attributable to acid generation. However, after treating T18, T29, and T39 strain extracts with catalase enzyme and performing anti-microbial tests against *Y. enterocolitica*, *S. mutans*, *E. coli*, and *S. aureus*, no bacterial extract was able to inhibit the growth of indicator pathogens, with the exception of strain T18 against *Y. enterocolitica* and *E. coli* and strain T39 against *S. aureus*. As a result, it was concluded that the inhibitory nature of those isolates against the mentioned pathogens is due to hydrogen peroxide production. Finally, the bacterial extracts were treated with protease K enzyme, and the antimicrobial properties of T18 and T39 strains were investigated against *Y. enterocolitica*, *E. coli*, and *S. aureus*. The results showed that no inhibition zone was observed, indicating that their extracts were bacteriocin-like against the mentioned pathogens.

Having acceptable inhibitory functions against pathogens is one of the most important intrinsic properties of probiotic bacteria. Probiotics belonging to the LAB group mainly inhibit the growth and spread of gram-positive and negative bacterial pathogens through a combination of various antimicrobial mechanisms such as the production and secretion of hydrogen peroxide (H_2_O_2_), organic acids, and inhibitory proteins (bacteriocin)^45^.

Our findings are supported by evidence indicating that the anti-pathogenic activities of LAB strains are primarily related to acidification capability and hydrogen peroxide secretion^20^. However, antagonistic activity due to bacteriocin secretion has been observed in a small number of gram-negative and positive pathogens^46^. These findings contradict previous research, which found that LAB bacteriocin are only effective against gram-positive pathogens and have no limiting effects on gram-negative pathogens due to their outer membranes^47,48^. The high antagonistic activities of LAB isolates against a wide variety of pathogenic bacteria have been described^49,50^, which are similar to our findings. Antibiotic resistance was found in some infections, such as *Y. enterocolitica* and *K. pneumoniae*, in addition to high prevalence. Antibiotic-resistant strains of these two gram-negative bacteria^51,52^ can thus be combated using LAB strains recovered from safe dairy sources^51,52^.

### Antibiotic susceptibility profiles

The antibiotic susceptibility results of selected LAB against nine clinically important and widely used antibiotics in Iran are presented in Table 4^53^. All three isolates were sensitive or semi-sensitive to cefixime, azithromycin, amoxicillin, doxycycline, cephalexin, and amoxicillin-clavulanic acid. High sensitivity to these antibiotics is probably due to the limited use of animal antibiotics in the rural area of Kermanshah province (sample collection areas).

In contrast to our results, the high resistance to cefixime, doxycycline, cephalexin, azithromycin, amoxicillin, and amoxicillin-clavulanic acid among the LAB bacteria was reported by other researches^54–56^. On the other hand, those isolates resisted to trimethoprim sulfamethoxazole, ciprofloxacin, and vancomycin. According to various reports, different strains of LAB group such as *Lactobacillus* genus carry the trimethoprim sulfamethoxazole, ciprofloxacin, and vancomycin resistance genes which recommend our results^57,58^. Overuse of antibiotics has led to the widespread emergence of antibiotic resistance genes in probiotic bacteria, which can transmit these genes to other microorganisms in the gastrointestinal tract and cause acute problems. Therefore, the susceptibility to antibiotics is a fundamental characteristic for their selection^59^.

### Adhesion ability to CaCo_2_ cells

In the present study, we have observed that strain T18 (69 ± 2.4%) exhibited the strongest adhesion to human intestinal CaCO_2_ cells, followed by strains T29 (51 ± 2.6%), T39 (47 ± 2.2%). Strains T3 and T46 showed no adhesion ability to human intestinal cells.

The capacity of probiotic microbes to bind to human intestinal epithelial cells is another characteristic for the choice of probiotics. In this way, attachment capacity considered as a standard for selecting a potential probiotic^60^. The adhesion mechanism is the interaction between lipids, peptidoglycans and surface proteins found on the bacterial cell wall. For several Lactobacillus species, bacterial cell wall-linked protein constituents promoting bacterial adhesion to intestinal epithelial cells have been shown^61^. Recently, several studies have indicated that a cell's hydrophobicity is correlated with the adhesion property of bacterial cells that favors colonization of epithelial cells^62^, as evidenced by our findings. Probiotic colonization of epithelial cells is thus strain-specific and relies in particular on the secretion of extracellular proteins by bacteria.

### Cholesterol assimilation

The cholesterol removal efficiency of strains from culture media is presented in Table 2. Strain T18 could assimilate 79% of cholesterol (118.5 out of 150 μg/mL) after 20 h incubation, while strain T29 and T39 could assimilate only 30 and 23% of cholesterol, respectively.

The hypocholesterolemic effect on host is another important but not essential features of probiotics. It has been reported that the hypocholesterolaemic effects of probiotics could be due to cholesterol assimilation or cholesterol binding to the cellular surface^63^. Probiotics have ability to lower cholesterol by several mechanisms^29^ such as conversion of cholesterol to coprostanol by reductase, cholesterol incorporation in the cell wall and disruption of cholesterol micelle in the intestine by deconjugated bile salts^1^.

### Hemolytic activity

All selected LAB strains produce no clear zone on the blood agar, which may indicate that the LAB strains were producing no β-hemolytic activity. Strains T18, T29, and T39 showed no hemolysis (γ-hemolysis), whereas stains T3 and T46 showed α-hemolytic activity. Thus, these two strains were deleted from further analysis. Similar results were reported in which most LAB showed no haemolytic activity. As per guidelines no hemolysis of blood cells proves the safety of probiotics (FAO/WHO, 2002).

### Auto-aggregation and co-aggregation

The results of cell auto-aggregation assay are shown in Table 2. The cell auto-aggregation rates of the strains ranged from 44 ± 1.2 to 79 ± 1.3%. The highest scores were obtained for strain T18, followed by T29 with 54 ± 1.1%. Furthermore, strain T39 showed the lowest auto-aggregation rates with 44 ± 1.2%.

The results of co-aggregation of selected strains in the presence of *E. coli*, *L. monocytogenes*, *Y. enterocolitica*, and *B. subtilis* separately at 37 °C for 4 h of incubation are shown in Table 5. The results showed that strain T18 exhibited the best co-aggregation ability. Co-aggregations of strain T18 with all four pathogens were higher (*p* ≤ 0.05) compared to strains T29 and T39.

**Table 5:** Co-aggregation (%) of selected strains with 4 pathogens during 4 h incubation at 37 °C.

Strains	Co-aggregation (%)			
	*E. coli* (PTCC 1276)	*L. monocytogenes* (ATCC 13932)	*Y. enterocolitica* (ATCC 2715)	*B. subtilis* (ATCC 19652)
T18	21.1 ± 1.39^a^	19.9 ± 1.44^a^	20.8 ± 1.92^b^	15.1 ± 1.74^a,b^
T29	7.1 ± 1.26^d,e^	5.6 ± 0.31^f,g^	7.1 ± 0.59^e^	5.6 ± 0.65^f^
T39	4.0 ± 0.21^g^	3.7 ± 0.31^g,h^	4.1 ± 0.36^f^	3.6 ± 0.59^g,h^

Values are mean ± standard deviation of triplicates.

^a–h^Means in the same column with different lowercase letters differed significantly (*p* ≤ 0.05).

The auto-aggregation and co-aggregation ability are two important features of probiotic bacteria, which are defined as the bacterial accumulation of the same species and of different species, respectively^64^. These two features are fundamental for probiotics because it seems that auto-aggregation is correlated with adherence to epithelial cells, while co-aggregation represents a defensive barrier for the colonization of pathogenic microorganisms^65^. Strain T18 showed the best hydrophobicity and adhesion ability to epithelial cells as well as the best co-aggregation ability. These results approve this opinion.

### Probiotic potato chips production, chemical and morphological analysis, and spraying efficiency

The moisture percentage of the turmeric and plain Tarkhineh pastes was high (73.40 and 73.81 g/100 g, respectively). Meanwhile, the pH of the Tarkhineh samples ranged from 4.83 to 4.94. Calcium and phosphorus concentrations in turmeric Tarkhineh were 326 and 268 mg/100 g, respectively, while plain Tarkhineh had 321 and 267 mg/100 g. For turmeric Tarkhineh, the crude protein, salt, ash, and fat contents were 4.14, 2.02, 2.45, and 1.04 g/100 g, respectively, and for plain Tarkhineh, they were 4.11, 2.01, 2.44, and 1.02 g/100 g. Finally, the carbohydrate content of turmeric and plain Tarkhineh paste was 16.35 g/100 g for turmeric and 16.02 g/100 g for plain Tarkhineh paste, respectively.

**Figure 1:**
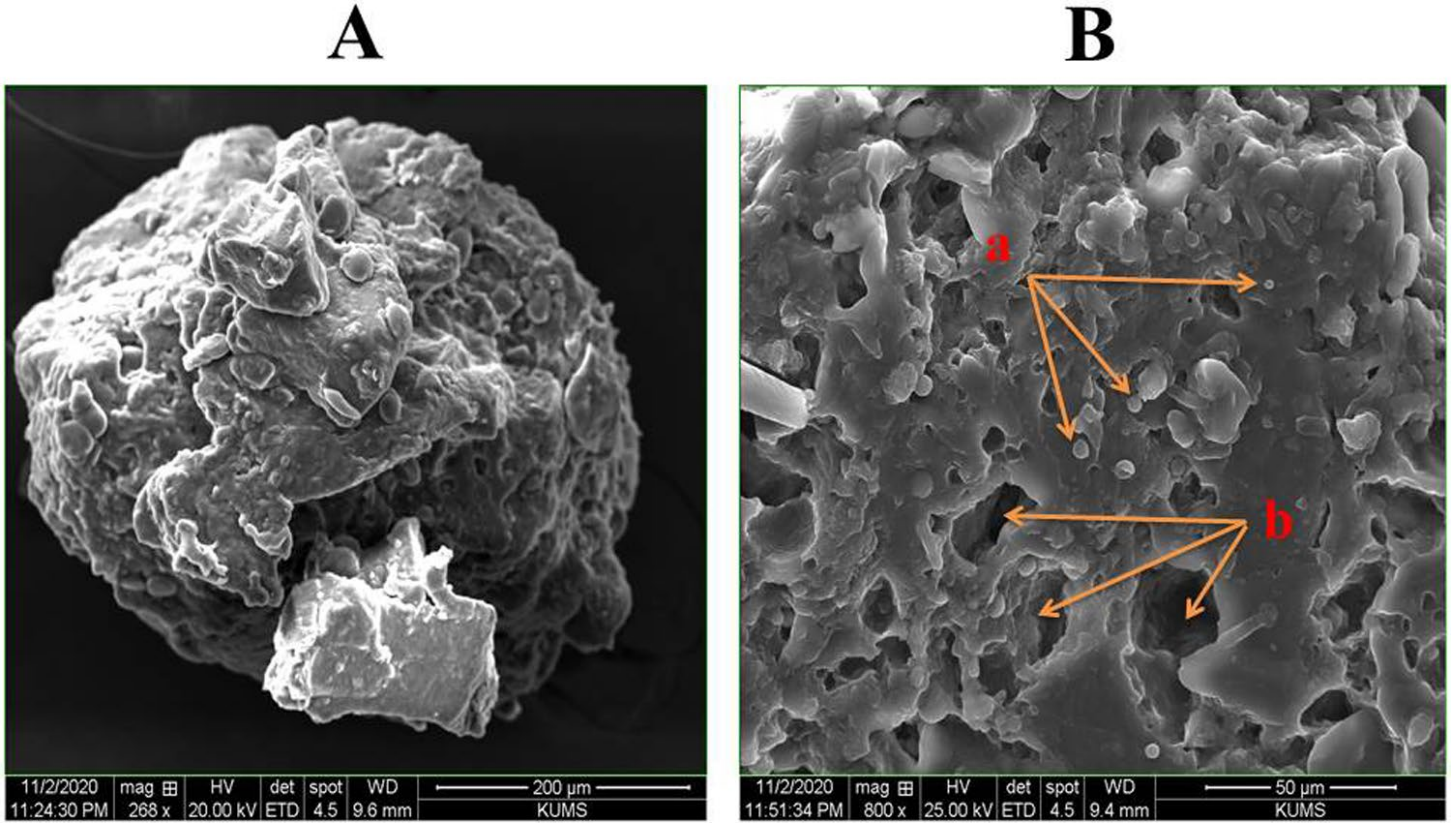
Morphological images of probiotic drop with Tarkhineh texture by the scanning electron microscopy (Hitachi SU3800): (**A**) spherical shape probiotic drop and their protective layers for the probiotic cells, (**B**): (a) network-like structures of Tarkhineh texture and entrapped probiotic cells and (b) tiny pores on the surface of Tarkhineh texture.

SEM morphological examination found that probiotic drops with varied Tarkhineh formulations had approximately elliptical to spherical shapes, with Tarkhineh texture acting as protective layers for probiotic cells (Fig. 1A). Furthermore, microscopy images revealed that Tarkhineh particles were integrated in network-like structures in probiotic drops and entrapped the probiotic cells (Fig. 1B(a)). Meanwhile, SEM images revealed a large number of small pores on the surface of all Tarkhineh formulations (Fig. 1B(b)).

To increase the storage stability and sensory qualities of the probiotic strain T18, two types of probiotic potato chip formulations (turmeric and plain Tarkhineh) were devised and developed. Low concentrations [less than 25% (w/v)] of Tarkhineh paste employed as a probiotic supportive companion resulted in reduced viscosity and did not produce homogeneous probiotic drops, according to studies. Due to its high viscosity, spraying Tarkhineh paste through a sprayer head at high concentrations [greater than 35 percent (w/v)] was challenging. As a result, the best concentration for the probiotic support matrix in the current investigation was 30 percent (w/v) Tarkhineh powder. Haghshenas et al.^35^ and Lotfipour et al.^34^ discovered similar probiotic drops with protective networks. Probiotic drops in spherical or elliptical shapes make industrial production easier and improve the look of probiotic goods^66,67^.

Probiotic drops had an average diameter of 460–740 m (based on 50 drops). The mean diameters of drops containing turmeric Tarkhineh were not statistically different (*p* ≤ 0.05) from drops containing plain Tarkhineh. This demonstrates that adding or removing turmeric has no effect on the size of probiotic drops. The small drop sizes in probiotic formulations, similar to our findings, do not alter the structure or texture of items and can be readily taken and recommended^68^. On the other hand, according to Sohail et al., the smaller sizes of probiotic drops (10–40 μm) were observed^69^. These high variations in sizes of drops can be due to different compositions and concentrations of probiotic companions^70^.

Trapped probiotic cells had a high spraying efficiency ranging from 98.2 to 99.6 percent. The results showed that bacterial cells were successfully entrapped (> 98%) in the prepared drops. According to the findings, no significant differences in spraying efficiency of probiotic cells were observed between these two Tarkhineh formulations; thus, spraying efficiency was formulation-independent. Because the minimum viable probiotic standard in food items is 10^6^ CFU/g, entrapped probiotic cells must have a high spraying efficiency in order to have health-promoting benefits. The efficient viable probiotic cells (> 10^8^ CFU/mL) can be released^71^ due to the high spraying efficiency (> 98%) observed in our results.

### Storage stability

**Figure 2:**
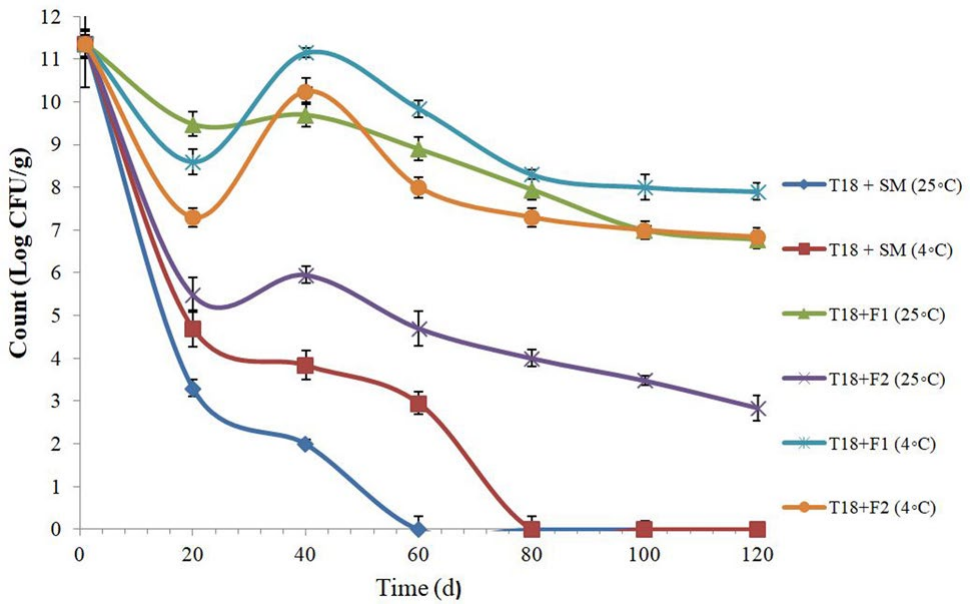
Storage stability of lyophilized and covered T18 probiotic strain with different Tarkhineh formulations during 120 days storage at 25 °C and 4 °C. T18: *Lactococcus lactis* T18. F1: plain Tarkhineh. F2: turmeric Tarkhineh. SM: 10% skim milk + 5% sucrose. Values shown are means ± standard deviations (n = 3).

Figure 2 shows the log CFU/g for lyophilized and coated T18 probiotic cells with various Tarkhineh formulations over storage period in potato chips. During four months of storage at 25 °C and 4 °C, lyophilized T18 cells showed a significant decline in cell viability. After 60 and 80 days of storage at 25 °C, the cell viability of lyophilized T18 cells decreased from 11.36 to 0.00 log CFU/g. The highest rates of reduction were detected in the first twenty days, while rates of reduction with a low slope were identified in the next one hundred days. This decreasing trend was most likely caused by the temperature shock in the first twenty days of storage and the subsequent adaptation process in the remaining days. Mostafa^17^ discovered the same results, with cell viability of lyophilized *Bifidobacterium longum* and *Lactobacillus helveticus* in potato chips dropping from 10.14 to 0.00 log CFU/g and 9.90 to 0.00 log CFU/g, respectively, after 2 months storage.

The covered T18 in those two Tarkhineh formulations (F1: plain Tarkhineh and F2: turmeric Tarkhineh) displayed significantly high cell viabilities (*p* ≤ 0.05) at the storage time at 25 °C and 4 °C. The T18 cells blend with turmeric Tarkhineh at 25 °C [T18 + F2 (25 °C)] had a week protection ability with 8.52 log decrease in CFU/g. Meanwhile, T18 cells blend with turmeric Tarkhineh at 4 °C [T18 + F2 (4 °C)] and blend with plain Tarkhineh at 25 °C [T18 + F1 (25 °C)] showed moderate protection abilities with around 4.52 and 4.58 log decrease in CFU/g respectively. But the excellent viability of covered cells was observed for T18 cells blend with plain Tarkhineh at 4 °C [T18 + F1 (4 °C)] with 3.46 log decrease in CFU/g (Fig. 2).

When compared to the higher temperature (25 °C), the probiotic formulations at the lower temperature (4 °C) had significantly superior protective powers (*p* ≤ *0.05*). Furthermore, the addition of turmeric to Tarkhineh formulations, particularly at 25 °C, reduced the viability of covered cells significantly (*p* ≤ 0.05) (Fig. 2).

Probiotic bacteria face numerous challenges when it comes to surviving in food products during storage. As a result, in order to solve this problem, appropriate storage conditions and protective settings must be chosen. Recent studies have used covering environments such as whey, skim milk, glucose, sucrose, and glycerol to protect probiotic cells^72^. Potato chips, for example, are typically stored at room temperature (25 °C) or in the refrigerator (4 °C) for at least 4 months. As a result, stability tests were carried out under the aforementioned conditions^23,73^. At 25 °C and 4 °C, probiotic cells in potato chips were counted every 20 days for up to 120 days.

Same as our results the high cell viability rates after storage at the low temperatures (4 °C) were observed for covered probiotics in prebiotic based hydrogels^16,74,75^. But, the viability of probiotic LAB cells used in potato chips is dropped dramatically after lyophilization process^23^. On the other hand, several researches same as our findings have proven the antimicrobial effects of turmeric^76,77^.

The dense network structure of probiotic drops and the high growth stimulation activities (prebiotic) of its ingredients (wheat semolina and buttermilk) can explain Tarkhineh-based formulations' excellent protective ability^78^. Higher Tarkhineh concentrations [> 30 percent (w/v)] resulted in denser network architectures and improved protection and growth in this study, although extrusion of Tarkhineh paste with high concentrations through sprayer heads was challenging and lowered production efficiency.

### Sensory evaluation

Potato chips' distinct qualities, such as taste, look, and flavor, have a significant impact on their production and marketability^79^. T18 probiotic formulations at refrigerated temperature exhibited acceptable protective properties based on storage stability data. As a result, sensory evaluation was carried out after 4 months of storage at 4 °C. Table 6 shows the mean values for sensory aspects as color, smell, appearance, texture, taste, and general approval for various probiotic potato chip formulations. Meanwhile, as a control, non-probiotic and commercial potato chip samples were chosen.

**Table 6:** The sensory evaluation (smell, color, taste, texture, appearance, and general acceptance) of non-probiotic potato chips, commercial potato chips, and probiotic potato chips with different prepared formulations (Tarkhineh) during 120 days storage at 4 °C. T18: *Lactococcus lactis KUMS-T18*. F1: plain Tarkhineh. F2: turmeric Tarkhineh. Values shown are means ± standard deviations (n = 3).

Formulation	Score designated (1–9)					
	Smell	Colour	Taste	Texture	Appearance	General acceptance
T18 + F1	7.00 ± 1.00^b^	5.87 ± 0.74^b^	6.00 ± 2.48^a^	7.07 ± 0.96^a^	6.73 ± 0.70^b^	7.53 ± 0.99^a^
T18 + F2	8.13 ± 0.74^a^	8.20 ± 0.86^a^	7.27 ± 1.28^a^	7.40 ± 0.83^a^	7.07 ± 0.88^ab^	7.87 ± 0.64^a^
Non-probiotic chips	5.00 ± 0.92^c^	6.13 ± 0.64^b^	5.67 ± 2.22^a^	6.93 ± 1.03^a^	6.80 ± 0.77^b^	5.73 ± 0.59^c^
Commercial chips	4.73 ± 0.80^c^	6.27 ± 0.59^b^	6.40 ± 2.80^a^	7.27 ± 0.70^a^	7.60 ± 0.63^a^	7.00 ± 1.07^b^

*Values are mean ± standard deviation of triplicates.

^a–c^Means in the same row with different lowercase letters differed significantly (*p* ≤ 0.05).

Sensory assessment scores comparing probiotic and control potato chips samples demonstrated a significant difference (*p* ≤ 0.05) at the end of storage time. According to the findings, probiotic potato chips with various formulations (plain Tarkhineh and turmeric Tarkhineh) received acceptable sensory evaluation scores when compared to control samples (Table 6).

The smell, color, and general acceptance scores of probiotic potato chips covered with plain Tarkhineh and turmeric Tarkhineh (T18 + F1 and T18 + F2) were the highest, but these sensory scores were the lowest in control samples (non-probiotic potato chips and commercial potato chips). However, there were no significant differences (*p* ≤ 0.05) between probiotic and non-probiotic samples in two sensory parameters (taste and texture) (Table 6).

The amazing sensory capabilities of Tarkhineh's fermentation process and the addition of spices like turmeric to its ingredients^80^ may explain the high overall scent, color, and general acceptance ratings of probiotic potato chips covered with Tarkhineh, specifically turmeric Tarkhineh formula (T18 + F2) throughout storage time. The considerable drop in sensory ratings in non-probiotic potato chips, on the other hand, could be attributed to a loss of flavor, odor, and color caused by the accumulation of peroxides and free fatty acids in the absence of viable probiotic cells over four months of storage^23^. As a result, it is concluded that the production of probiotic potato chips coated with Tarkhineh is technically feasible and cost-effective due to their high ability to maintain quality and sensory properties during shelf life.

### Molecular identification and probiotic characterization

The PCR-amplified fragments of the 16S-rRNA genes of the selected strains were sequenced. Based on results, isolate T18 belonged to *Lactococcus lactis* (Accession No. MW429822), isolate T29 belonged to *Enterococcus hirae* (Accession No. MW433682) and isolate T39 belonged to *Enterococcus mundtii* (Accession No. MW433679).

## Conclusion

Among the tested LAB, strain T18, *Lactococcus lactis* as a safe strain, had the highest probiotic scored properties such as tolerance to low pH and high bile salt, anti-pathogenic activity, hydrophobicity, adhesion to human epithelial cells, auto- and co-aggregation, cholesterol assimilation and antibiotic susceptibility. Therefore, this strain can prevent infectious diseases and control oxidative stress related to excessive consumption of potato chips. On the other hand, two Tarkhineh formulations were compared to evaluate their suitability for spraying the newly isolated probiotic strain and assess its protective effect and sensory properties in potato chips.

Plain Tarkhineh was the most suitable texture for protecting *Lactococcus lactis* throughout four months period time at 4 °C as it preserved the probiotic cell viability at 8 × 10^7^ CFU/g. Meanwhile, it displayed high overall smell, color, and general acceptance scores during storage time. Potato chips containing *Lactococcus lactis* blended with traditional fermented dairy products such as Tarkhineh showed promising features to meet consumer expectations and demands. In conclusion, we recommend that such an invented product be consumed between meals and in appropriate amounts to benefit from the properties of probiotics and dairy products, as well as the minerals and vitamins needed by the body.
